# Pregabalin for Refractory Radicular Leg Pain due to Lumbar Spinal Stenosis: A Preliminary Prospective Study

**DOI:** 10.1155/2016/5079675

**Published:** 2016-03-29

**Authors:** Sumihisa Orita, Masaomi Yamashita, Yawara Eguchi, Miyako Suzuki, Gen Inoue, Masayuki Miyagi, Tomoko Watanabe, Tomoyuki Ozawa, Hiroto Kamoda, Tetsuhiro Ishikawa, Yasuchika Aoki, Toshinori Ito, Go Kubota, Munetaka Suzuki, Kazuyo Yamauchi, Eiji Hanaoka, Yoshihiro Sakuma, Jun Shimbo, Yasuhiro Oikawa, Takane Suzuki, Kazuhisa Takahashi, Seiji Ohtori

**Affiliations:** ^1^Department of Orthopaedic Surgery, Graduate School of Medicine, Chiba University, Chiba 260-8670, Japan; ^2^Department of Orthopaedic Surgery, Japan Community Health Care Organization Funabashi Central Hospital, Funabashi 273-0021, Japan; ^3^Department of Orthopaedic Surgery, Shimoshizu National Hospital, Yotsukaido 284-0003, Japan; ^4^Department of Orthopaedic Surgery, Kitasato University, Sagamihara 252-0375, Japan; ^5^Department of Orthopaedic Surgery, Tsuga Saito Orthopaedic Clinic, Chiba 264-0025, Japan; ^6^Department of Orthopaedic Surgery, Showa University, Tokyo 142-8666, Japan; ^7^Department of Orthopaedic Surgery, Chiba Cancer Center, Chiba 260-0801, Japan; ^8^Department of Orthopaedic Surgery, Sanmu Medical Center, Sanmu 289-1326, Japan; ^9^Department of Orthopaedic Surgery, Eastern Chiba Medical Center, Togane 283-8686, Japan; ^10^Department of Orthopaedic Surgery, Kumagaya General Hospital, Kumagaya 360-0013, Japan; ^11^Department of Orthopaedic Surgery, Japan Community Health Care Organization Chiba Hospital, Chiba 260-8710, Japan; ^12^Department of Orthopaedic Surgery, Chiba National Medical Center, Chiba 260-0042, Japan; ^13^Department of Orthopaedic Surgery, Funabashi Municipal Medical Center, Funabashi 273-8588, Japan

## Abstract

We investigated the efficacy of pregabalin (PGB) for neuropathic leg pain in lumbar spinal stenosis (LSS) patients with disturbed activities of daily living (ADL)/quality of life (QOL) in a prospective observational study. Subjects were a total of 104 LSS patients with neuropathic pain (NeP) in leg and neurological intermittent claudication (IMC) refractory to nonsteroidal anti-inflammatory drugs (NSAIDs) for at least a month. NeP was identified using screening tool, Pain DETECT questionnaire. Visual analog scale (VAS) scores and responses to the Japanese Orthopaedic Association Back Pain Evaluation Questionnaire (JOABPEQ) were assessed before and 6 weeks after PGB treatment initiation. Changes in IMC distance and adverse events were also recorded. PGB significantly improved their VAS scores for pain and sleep quality (*P* < 0.001). With respect to JOABPEQ, significant improvements were observed with regard to the following dimensions: pain-related disorders (*P* < 0.01), lumbar spine dysfunction (*P* = 0.031), gait disturbance (*P* = 0.028), and psychological disorders (*P* = 0.014). The IMC distance showed an improvement tendency after PGB treatment, albeit with no significance (*P* = 0.063). Minor adverse events such as dizziness were observed. PGB can be effective for neuropathic leg pain refractory to NSAIDs in LSS patients, resulting in not only pain control but also improving lower back pain-related ADL/QOL scores.

## 1. Introduction

Patients with lumbar spinal stenosis (LSS) often experience chronic and refractory pain, and this condition is the most common reason for spinal surgery in patients older than 65 years [1]. The major pathologies underlying pain in LSS patients are neurological damage to the cauda equina and spinal nerve compression by the degenerative and bulged intervertebral disc, degenerative facet joint, and hypertrophic bone and/or ligamentum flavum. Such patients experience various forms of pain, including low back pain (LBP), radicular leg pain, and intermittent claudication (IMC); the lattermost is characterized by increasing lower limb pain, numbness, and gait disturbance.

Patients with LSS exhibit accumulated neuronal damage, which results in chronic pain that is refractory to existing analgesics such as nonsteroidal anti-inflammatory drugs (NSAIDs) because it originates from the damaged nervous system known as neuropathic pain (NeP), along with nociceptive pain (NocP) [2]. In patients with LSS, severe damage to the cauda equina and/or spinal nerve causes radicular leg pain, with NeP accounting for 56.9% of such cases [3]. Furthermore, patients with LSS develop other neurological disorders, including IMC, which is a neuropathic manifestation of chronically compressed and ischemic nerve roots. Because of this accumulated and irreversible neuronal damage, NeP is occasionally difficult to treat with the conventional analgesics used to treat reversible NocP.

Pregabalin (PGB; (S)-3-(aminomethyl)-5-methylhexanoic acid), is a newer generation gabapentinoid that was originally synthesized as an adjuvant antiepileptic drug over four decades ago. Since 2004, however, it has been commonly used as a first-line drug for NeP in the United States and Europe [4, 5]. This drug alleviates NeP by suppressing the secretion of pain-related mediators in the synapses through binding to the *α*_2_ *δ* subunits of Ca^2+^ channels [6]. The effectiveness of PGB in patients with epilepsy, posttherapeutic neuralgia, and diabetic neuropathy has already been established, and one previous study has reported its potential usefulness for the control of LSS-related neuropathic leg pain [7, 8]. However, no study has evaluated the effects of this drug on parameters other than pain, including activities of daily living (ADL) and quality of life (QOL), in LSS patients.

On the basis of the above perspectives, the current study evaluated the effects of PGB on NeP in the legs, ADL, and QOL in patients with LSS.

## 2. Materials and Methods

### 2.1. IRB Approval and Inclusion Criteria

This prospective, observational study was approved by our Institutional Review Board and is registered in the Japanese national clinical trials registry (UMIN000017743, evidence II). Informed consent was obtained from all patients before enrollment. In total, 19 certified Japanese orthopaedic surgeons from 13 facilities were involved in this study.

PGB-naïve LSS patients with low back and leg pain aged 20–90 years who were diagnosed with chronic NeP using the Pain DETECT questionnaire (PD-Q), which is a patient-oriented and validated screening tool, were included in this study [9] (Table 1). A PD-Q score of 19–38, 0–12, and 13–18 indicated NeP, NocP, and mixed pain (NeP and NocP), respectively; the patients included in this study exhibited a score of ≥13. The minimum duration of LSS, which was diagnosed using magnetic resonance imaging (MRI), was 6 months, and all patients mainly experienced leg pain (visual analog scale (VAS) score ≥ 30 (mm); 0 = no pain, 100 = worst possible pain) and IMC that were refractory to NSAIDs administered for at least a month. Sleeping disorders were also evaluated using VAS (0 = no sleep at all, 100 = best possible sleep). An adequate creatinine clearance of ≥60 mL/min was confirmed in all patients. With regard to radiological evidence and the pathology underlying LSS, a mixed pathology (compression of both the spinal nerve and cauda equina) accounted for approximately 50% patients in each set, followed by spinal nerve compression only and cauda equina compression only.

### 2.2. Exclusion Criteria

Patients with conditions that could alter their pain perceptions, such as diabetes mellitus, psychiatric disorders, and chronic regional pain syndrome (CRPS), were excluded, as were patients with a history of previous spine surgeries for LSS, nerve block injection within 2 months before trial registration, peripheral vascular disorder, and malignant tumor within 2 years before trial registration. Patients with a fresh vertebral fracture or spinal tumor identified on MRI were also excluded.

### 2.3. Intervention and Evaluation

**Figure 1:**
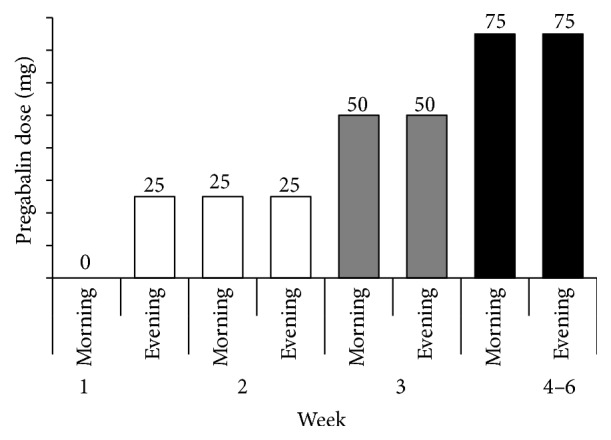
Pregabalin (PGB) treatment schedule. Treatment is initiated with the minimum dose (25 mg) administered in the evening, followed by a gradual increase. The maximum dose is 150 mg/day depending on the pain intensity.

Following appropriate NSAID treatment for more than a month, the patients were additionally prescribed PGB without discontinuing or modifying the dose of NSAIDs. Treatment was initiated with a dose of 25 mg/d, which was gradually increased every week to 150 mg/d over 6 weeks, depending on their pain intensity, until a VAS score of <30 was achieved (Figure 1).

The primary endpoint was the direct analgesic effect of PGB on leg pain evaluated using VAS, while the secondary endpoints comprised changes in circumstantial aspects of the pain, namely, sleep quality and ADL/QOL. For the evaluation of ADL/QOL, the Japanese Orthopaedic Association Back Pain Evaluation Questionnaire (JOABPEQ) was used, while VAS was used to assess sleep quality. JOABPEQ is an ensured and a validated method for evaluating the degree of LBP-related ADL/QOL and includes the following five dimensions: pain-related disorders (PaD), gait disturbance (GaD), lumbar spine dysfunction (LuD), social life disturbance (SoD), and psychological disorders (PsD) [10]. The minimum score is 0 and the maximum score is 100; a higher score indicates a better health status. All evaluations were conducted before (baseline) and 6 weeks after PGB initiation. We also recorded changes in the IMC distance and adverse events during PGB administration. IMC distance was provided by the numbers of maximum round-trips of the distinct distance standards in each clinic, such as from parking to the clinic.

Statistical analyses were performed by a biostatistician using the SAS software ver. 9.2 (SAS Inc., Cary, NC). Data were analyzed using a combination of unpaired 2-tailed* t*-tests, Fisher's exact test, and multivariate linear regression analyses. A *P* value of <0.05 was considered statistically significant.

## 3. Results

### 3.1. Patient Selection and Demographics

**Figure 2:**
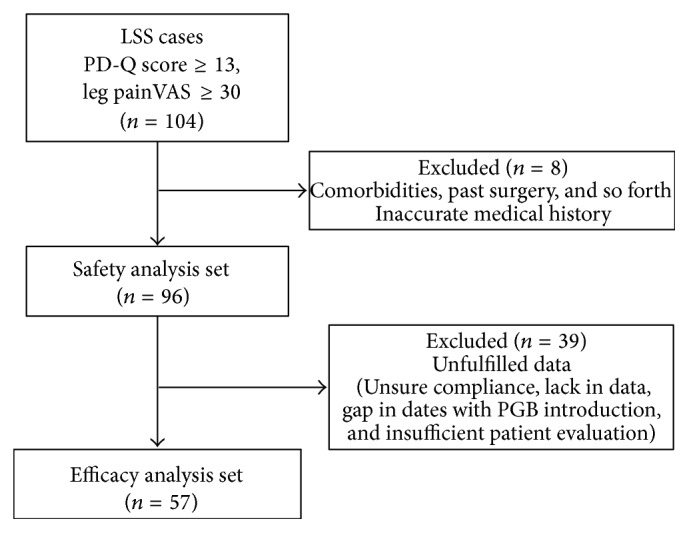
Patient selection flowchart. LSS: lumbar spinal stenosis; PD-Q: Pain DETECT questionnaire; VAS: visual analog scale; PGB: pregabalin.

Figure 2 shows the flowchart for patient selection. A total of 104 LSS patients satisfied the inclusion criteria, that is, a PD-Q score of ≥13 and a VAS score of ≥30 for leg pain. From these, eight patients with comorbidities such as diabetes and peripheral vascular disease or an inaccurate medical history were excluded. The remaining 96 patients were included in safety analysis for PGB-related adverse events. Subsequently, 39 patients with insufficient data, gap in date (≥2 weeks) with PGB introduction, and/or insufficient evaluations were excluded. Finally, 57 patients were included in the efficacy analysis.

Table 2 shows the demographic data of patients. The average disease duration was 38.2 ± 36.6 (mean ± standard deviation (SD)) months in the safety analysis set and 45.4 ± 42.7 months in the efficacy analysis set; the difference was not significant (*P* = 0.67). There were no significant differences in demographic characteristics such as sex, age, and physique (height and weight) between the two sets. LSS pathology showed no significance among the pathologies, spinal nerve compression, cauda equina compression, or both (*P* = 0.82).

### 3.2. VAS Score Changes

**Figure 3:**
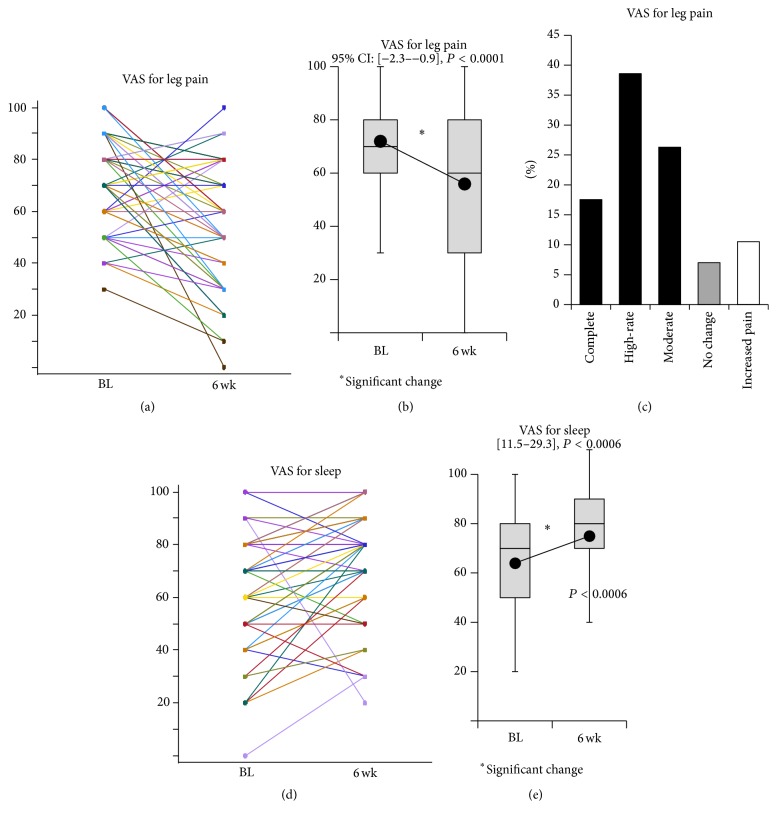
Visual analog scale (VAS) scores. (a, b, and c) Leg pain; raw data, followed by box and whisker plot. (d, e) Sleep quality; box and whisker plot. The average score at each time point is connected to indicate the tendency for change. Both scores are significantly decreased after the 6-week administration of pregabalin (PGB).

Figure 3 shows the changes in VAS scores for leg pain and sleep quality; both showed a significant decrease after the 6-week administration of PGB (95% confidence interval (CI), −2.3–−0.9; *P* < 0.0001 and 11.5–29.3; *P* = 0.0006, resp.). The scores were not influenced by the LSS pathology.

Figure 3(c) shows the categorical presentation of the PGB administration; complete relief (VAS 0) was achieved in 10 patients out of 57 patients (17.5%), high-rate relief (VAS 10–20) was achieved in 22 patients (38.6%), and moderate relief (VAS 30–40) was achieved in 15 patients (26.3%) while there were 4 patients with no change (7.0%) and 6 patients with increased pain (10.5%).

### 3.3. JOABPEQ Score Changes

**Figure 4:**
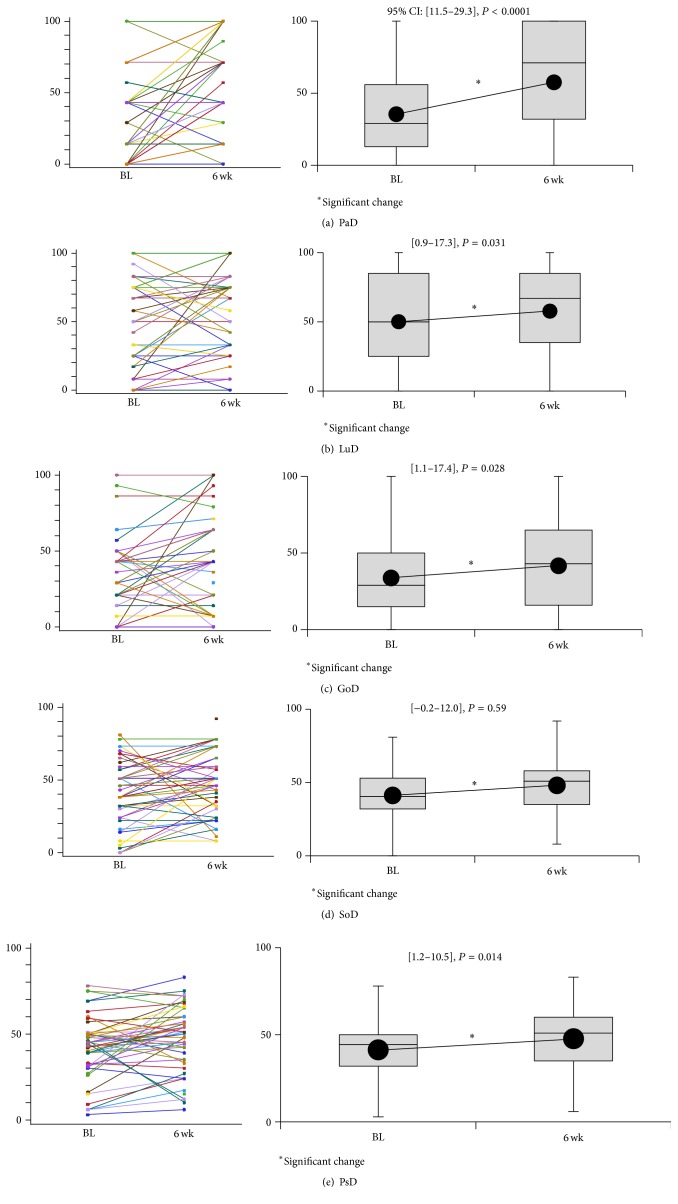
Score changes of the Japanese Orthopaedic Association Back Pain Evaluation Questionnaire (JOABPEQ) scores at baseline and at 6 weeks after pregabalin initiation. The average score at each time point is connected to indicate the tendency for change. Each of the items consists of raw data, followed by box and whisker plot. PaD: pain-related disorders, GaD: gait disturbance, LuD: lumbar spine dysfunction, SoD: social life disturbance, and PsD: psychological disorders. The scores for PaD, LuD, GaD, and PsD show a significant improvement after PGB treatment (identified by an asterisk), while that for SoD shows an improvement tendency with no significance.

Figure 4 shows the JOABPEQ scores at baseline and 6 weeks after PGB initiation. The scores for PaD (95% CI, 11.5–29.3; *P* < 0.0001), LuD (95% CI, 0.9–17.3; *P* = 0.031), GaD (95% CI, 1.1–17.4; *P* = 0.028), and PsD (95% CI, 1.2–10.5; *P* = 0.0142, *P* = 0.014) showed a significant improvement after PGB treatment, while that for SoD showed an improvement tendency with no significance (95% CI, −0.2–12.0; *P* = 0.059). The scores were not influenced by the LSS pathology.

### 3.4. IMC Distance Changes

**Figure 5:**
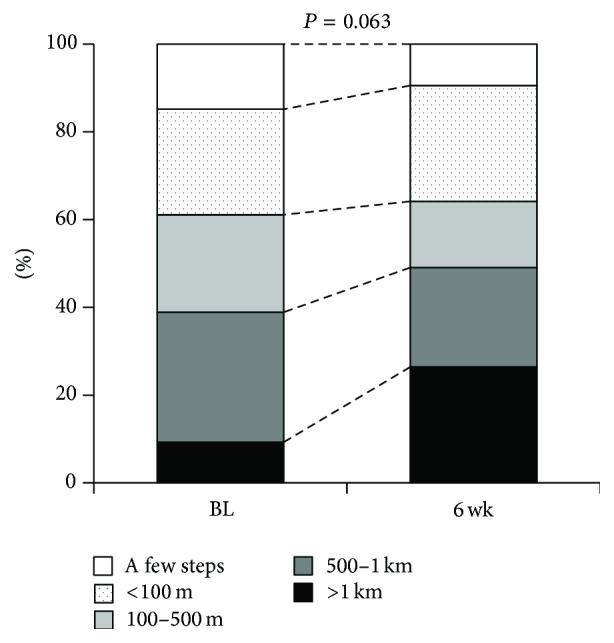
Changes in the intermittent claudication (IMC) distance. The IMC distance shows a tendency to increase after the 6-week administration of PGB, with apparent significance.

The IMC distance showed a tendency to increase after the 6-week administration of PGB, although the change was not significant (*P* = 0.063; Figure 5). The LSS pathology did not influence the changes.

### 3.5. Adverse Events

Among the 96 patients in the safety analysis set, 12 (12.5%) experienced PGB-related adverse events (Table 3), which included ataxia (*n* = 3, 3.1%), dizziness (*n* = 2, 2.1%), nausea (*n* = 2, 2.1%), and edema (*n* = 1, 1.0%; Table 3). PGB was withdrawn for 10 of the 12 patients, while the dose was decreased for the remaining two. There was no correlation between the PGB dose and withdrawal rate. All patients recovered without any special treatment.

## 4. Discussion

The current study evaluated the therapeutic effects of PGB in patients with LSS accompanied by neuropathic leg pain and IMC refractory to NSAIDs. PGB significantly improved the VAS scores for leg pain and sleep quality and the JOABPEQ scores for PaD, LuD, GaD, and PsD (*P* < 0.05). The IMC distance showed a tendency to improve, albeit with no significance. Finally, PGB-related adverse events such as dizziness, ataxia, and somnolentia, which were of mild severity and resolved without any additional treatment, were observed. First we should keep in mind that the current preliminary study has relative control of the patients themselves with a limited observational period, which can contain placebo effect.

### 4.1. Effects of PGB on LSS-Related Leg Pain

A VAS score of ≥30 is considered to represent moderate pain according to the distribution of VAS scores corresponding to a four-point categorical scale (none, mild, moderate, and severe) defined in a previous study [11]. Therefore, the patients included in the present study were considered to have moderate-to-severe leg pain with a mean VAS score of 72, which significantly improved after PGB treatment for 6 weeks. There are multiple pathological mechanisms underlying leg pain in LSS patients; central canal stenosis may compress the cauda equina, whereas lateral recess stenosis and foraminal stenosis may compress the nerve roots while sparing the spine, resulting in inflammation, ischemia, malnutrition, nerve degeneration, nerve injury, and mechanoreceptive compression [1]. This implies the involvement of both NocP and NeP. Significant clinical signs and symptoms of NeP include frequent radicular pain radiating beyond the knee toward the foot (40.0%), a positive Lasegue sign (18.4%), and/or an absent patellar reflex (17.3%), and a third of patients with neuropathic leg pain show these symptoms [12]. All the patients included in the present study also exhibited these signs and symptoms.

Taken together, the effects of PGB on neuropathic leg pain in the LSS patients in the current study were similar to those observed in a previous study including 96 LSS patients [1], thus confirming the analgesic effects of PGB in LSS patients with neuropathic leg pain. Total of 82.4% of the patients showed improved pain, which indicates clinical efficacy of PGB.

### 4.2. Effects of PGB on JOABPEQ Scores and IMC Distance

The current study suggests that PGB not only alleviates NeP but also improves the associated symptoms such as sleep disturbance, mood disorders, and disability; this eventually translates into an improvement in overall health. JOABPEQ is a new outcome measure that enables the evaluation of dysfunction, disabilities, and psychosocial problems caused by a disease on the basis of the Roland-Morris Disability Questionnaire and Short-Form (SF) 36 [10, 13]. Among the five dimensions, the scores for LuD, GaD, and SoD are reported to decline with age [13], although this was not observed in our study.

There may be a correlation between the improvement in the JOABPEQ score for PsD and that in the VAS score for sleep quality, because the two factors are clinically related and because PGB itself can alleviate anxiety, depression, and sleep disturbance [14–18]. However, the efficacy of PGB is lower than that of benzodiazepines, with a low tolerance to dependence [19].

Interestingly, we observed a significant change in the score for GaD and an insignificant improvement in the IMC distance, even though the two parameters are believed to be closely related. Evidence regarding the effects of PGB on IMC is somewhat controversial. Markman et al. reported that PGB did not increase the walking duration of LSS patients during a treadmill test [20]. However, the study was performed in a controlled environment under limited conditions and involved PGB administration for 13 days. On the other hand, the current study also included QOL and ADL evaluations using JOABPEQ. The tendency for an improvement in the IMC distance is an important finding with regard to the significant improvement in the score for GaD, which is closely associated with IMC. No previous study has evaluated the relationship between these two parameters, necessitating further studies with a larger number of subjects. However, IMC distance was self-evaluated in the current study, and it could have been affected by aspects such as weather, temperature, and walking duration. This was probably the reason for the insignificant change in the IMC distance. A significant change in the score for GaD can support the change in the IMC distance through objective and validated JOABPEQ scoring. Future studies should include more objective measurements such as a treadmill test to increase the statistical accuracy of the results.

A clinical study based on patient-oriented outcome measures such as the SF-Mc Gill Pain Questionnaire and EuroQol 5D showed the effectiveness of PGB in alleviating painful lumbar radiculopathy [21]. The strength of the current study is that it evaluated not only pain but also ADL and QOL using JOABPEQ, which is a patient-oriented and validated measure, sleep quality, and IMC distance.

### 4.3. Adverse Events

PGB-associated adverse events occurred in 12.5% patients, including dizziness, nausea, ataxia, somnolentia, weight gain, and peripheral edema. These are common PGB-related events, and their intensity generally increases with an increase in the PGB dose [22]. The incidence of adverse events was 18.8% in the safety analysis set, which is similar to the reported incidence [22], and there was no association between the PGB dose and withdrawal rate. A previous retrospective study [23] reported that the duration of therapy for somnolentia, NSAID prescription, age, maintenance dose for ataxia, serum creatinine level for body weight gain, neurotrophin use, and serum creatinine level for edema, which may go unrecognized and lead to the loss of mobility and prolonged hospitalization [24], were risk factors for PGB-related adverse events. These adverse events may be caused by increased inadvertent actions of PGB [25], which inhibits the various types of calcium channels in different brain areas and results in impairment/decreased activity in higher cortical functions, thus causing dizziness, ataxia, and cognitive impairment [22], all of which can affect ADL. These adverse events sometimes necessitate PGB withdrawal.

### 4.4. Limitations

The current study has some limitations. First, it was not a randomized controlled, blinded study. Also the current study allowed drop-out data due to unsatisfied data collection under strict control, which should be improved more aggressively. Second, it lacked a strict control; the status of refractory pain before PGB treatment was used as a relative control. This may have led to potential cross-interaction between PGB and preprescribed drugs, although PGB reportedly does not interact with other drugs [5]. Further studies with a strict placebo control are necessary. Third, patients were not assessed by sex and/or age. The prevalence of degenerative lumbar disease, which causes LSS, can vary according to sex and age, and this can affect the results of JOABPEQ [13]. Also it should evaluate more objective standards such as Shuttle Walking Test, Oxford Spinal Stenosis Score, and Zurich Claudication Questionnaire/Swiss Spinal Stenosis Questionnaire for LSS. Further studies should address this issue. Fourth, the observational period of six weeks is relatively short. The period was set to avoid possible spontaneous recovery with longer observational period, which can affect the result of the current study. Longer observational study should be designed in the future study. Lastly, the IMC distance should be measured using more objective functional measurement method. This was difficult to achieve in all of the involved facilities, which should be one of the reasons for the nonsignificance in the IMC distance.

The major advantages of PGB include its relative reliability, ease of use, and high tolerability [26]. Thus far, it has been used for NeP associated with alterations in both the central nervous system (e.g., spinal cord injury) [23] and peripheral nervous system (postoperative pain) [27–32], and the current study proved that it is also effective in alleviating neuropathic leg pain and improving ADL/QOL, including sleep quality, in LSS patients. These findings indicate that persistent leg pain in LSS patients that is refractory to NSAIDs can be effectively controlled using PGB, which is initiated with a low dose that is gradually increased. Other surgeons have already reported the efficacy of the combined administration of cyclooxygenase-2 inhibitors and PGB for pain control after arthroplasty [33–35].

In conclusion, the results of this study suggest that PGB can not only control neuropathic leg pain refractory to NSAIDs but also affect lumbar spinal function, gait disturbances, psychological disorders, and sleep quality. There is a possibility that an appropriate diagnosis of NeP and early PGB initiation can significantly decrease medical costs by avoiding prolonged treatment [36].

## Figures and Tables

**Table 1:** Pain DETECT questionnaire [9].

Item	Score
*Gradation of pain* ^*∗*^	
(i) Do you suffer from a burning sensation (e.g., stinging nettles) in the marked areas?	0–5
(ii) Do you have a tingling or prickling sensation in the area of your pain (like crawling ants or electrical tingling)?	0–5
(iii) Is light touching (clothing. a blanket) in this area painful?	0–5
(iv) Do you have sudden pain attacks in the area of your pain, like dectric shocks?	0–5
(v) Is cold or heat (bath water) in this area occasionally painful?	0–5
(vi) Do you suffer from a sensation of numbness in the areas that you marked?	0–5
(vii) Does slight pressure in this area, for example, with a finger, trigger pain?	0–5
*Pain course pattern*	
Please select the picture that best describes the course of your pain:	
Persistent pain with slight fluctuations	0
Persistent pain with pain attacks	−1
Pain attacks without pain between them	+1
Pain attacks with pain between them	+1
*Radiating pain*	
Does your pain radiate to other regions of your body? Yes/No	

^*∗*^For each question: never noticed, 0; hardly noticed, 1: slightly noticed, 2: moderately noticed, 3; strongly noticed, 4; very strongly noticed, 5.

**Table 2:** Patient demographics.

		Safety analysis (*n* = 96)	Efficacy analysis (*n* = 57)	*P*
Sex (M/F)		46/50	22/35	0.76
Age (years; mean ± SD)		69.1 ± 10.6	71.0 ± 8.7	0.85
Height (cm; mean ± SD)		158.7 ± 9.2	156.9 ± 8.3	0.66
Weight (kg; mean ± SD)		58.7 ± 10.8	57.4 ± 10.7	0.85
LSS duration (months)		38.2 ± 36.6	45.4 ± 42.7	0.67

Stenosis severity on MRI, *n* (%)	Mild	24 (25.3)	9 (15.8)	0.55^*∗*^
	Moderate	42 (44.2)	25 (43.9)	
	Severe	29 (30.5)	23 (40.4)	

LSS pathology, *n* (%)	Spinal nerve compression	34 (36.2)	16 (28.6)	0.82^*∗*^
	Cauda equina compression	10 (10.6)	7 (12.5)	
	Both (mixed)	50 (53.2)	33 (58.9)	

^*∗*^Significantly different according to chi-square and Fisher's tests.

MRI: magnetic resonance imaging; SD: standard deviation; LSS: lumbar spinal stenosis.

**Table 3:** Adverse events.

Age/sex	Event	PGB dose at onset (mg/day)	Onset (days after PGB prescription)	Severity^*∗*^	Causal association	PGB prescription	Special treatment
76/M	Rash	25 mg × 1 (25)	5	Mild	Unclear	Withdrawn	No
76/F	Dizziness	25 mg × 2 (50)	14	Mild	Yes	Withdrawn	No
79/M	Nausea	50 mg × 2 (100)	30	Mod.	Yes	Withdrawn	No
79/M	Ataxia	50 mg × 2 (100)	30	Mod.	Yes	Withdrawn	No
79/M	Somnolentia	25 mg × 2 (50)	10	Mild	No	Dose decreased	No
81/M	Increased numbness	75 mg × 2 (150)	25	Mod.	Unclear	Withdrawn	No
82/M	Increased numbness	25 mg × 1 (25)	14	Mod.	Unclear	Withdrawn	No
78/F	Fever	75 mg × 2 (150)	33	Mod.	Unclear	Withdrawn	No (blood test)
78/F	Nausea	75 mg × 2 (150)	33	Mod.	Unclear	Withdrawn	No
64/F	Ataxia	50 mg × 2 (100)	14	Mod.	Yes	Dose decreased	No
64/F	Dizziness	50 mg × 2 (100)	14	Mod.	Yes	Dose decreased	No
64/F	Nausea	50 mg × 2 (100)	14	Mod.	Yes	Dose decreased	No
64/M	Rash	25 mg × 1 (25)	6	Mod.	Unclear	Withdrawn	No
71/F	Somnolentia	25 mg × 2 (50)	16	Mild	Yes	Continued	No
71/F	Ataxia	25 mg × 2 (50)	16	Mild	Yes	Continued	No
54/F	Weight gain	—	231	—	Unclear	Withdrawn	—
73/F	Edema	—	140	Mild	Yes	Dose decreased	No
74/M	Edema	150 mg × 2 (300)	35	Mild	Yes	Dose decreased	No

^*∗*^Mod.: moderate.
